# Misoprostol for postpartum hemorrhage prevention at home birth: an integrative review of global implementation experience to date

**DOI:** 10.1186/1471-2393-13-44

**Published:** 2013-02-20

**Authors:** Jeffrey Michael Smith, Rehana Gubin, Martine M Holston, Judith Fullerton, Ndola Prata

**Affiliations:** 1 Jhpiego, 1776 Massachusetts Ave., NW#300, Washington, DC 20036, USA; 2 Jhpiego, 1615 Thames St. #300, Baltimore, MD 21231, USA; 3 Venture Strategies Innovations, 2115 Milvia St., Suite 4A, Berkeley, CA 94704, USA; 4 University of California, San Diego (Ret), 7717 Canyon Point Lane, San Diego, CA 92126, USA; 5 School of Public Health, University of California, Berkeley, 229 University Hall, Berkeley, CA 94720-6390, USA

**Keywords:** Community-based distribution mechanisms, Misoprostol, Coverage, Safety, Serious adverse events, Home birth, Postpartum hemorrhage

## Abstract

**Background:**

Hemorrhage continues to be a leading cause of maternal death in developing countries. The 2012 World Health Organization guidelines for the prevention and management of postpartum hemorrhage (PPH) recommend oral administration of misoprostol by community health workers (CHWs). However, there are several outstanding questions about distribution of misoprostol for PPH prevention at home births.

**Methods:**

We conducted an integrative review of published research studies and evaluation reports from programs that distributed misoprostol at the community level for prevention of PPH at home births. We reviewed methods and cadres involved in education of end-users, drug administration, distribution, and coverage, correct and incorrect usage, and serious adverse events.

**Results:**

Eighteen programs were identified; only seven reported all data of interest. Programs utilized a range of strategies and timings for distributing misoprostol. Distribution rates were higher when misoprostol was distributed at a home visit during late pregnancy (54.5-96.9%) or at birth (22.5-83.6%), compared to antenatal care (ANC) distribution at any ANC visit (22.5-49.1%) or late ANC visit (21.0-26.7%). Coverage rates were highest when CHWs and traditional birth attendants distributed misoprostol and lower when health workers/ANC providers distributed the medication. The highest distribution and coverage rates were achieved by programs that allowed self-administration. Seven women took misoprostol prior to delivery out of more than 12,000 women who were followed-up. Facility birth rates increased in the three programs for which this information was available. Fifty-one (51) maternal deaths were reported among 86,732 women taking misoprostol: 24 were attributed to perceived PPH; none were directly attributed to use of misoprostol. Even if all deaths were attributable to PPH, the equivalent ratio (59 maternal deaths/100,000 live births) is substantially lower than the reported maternal mortality ratio in any of these countries.

**Conclusions:**

Community-based programs for prevention of PPH at home birth using misoprostol can achieve high distribution and use of the medication, using diverse program strategies. Coverage was greatest when misoprostol was distributed by community health agents at home visits. Programs appear to be safe, with an extremely low rate of ante- or intrapartum administration of the medication.

## Background

The lifetime risk of dying from pregnancy or childbirth ranges from about one in 39 in sub-Saharan Africa to 1 in 3800 in developed countries [1]. Hemorrhage continues to be one of the leading causes of maternal death in developing countries, and the predominant cause in Africa (34%) and Asia (31%) [2,3]. Postpartum hemorrhage (PPH), defined as blood loss ≥ 500 mL, occurs in approximately 6% of deliveries globally and severe PPH (≥ 1000 mL) in an additional 1.8%, with wide variation across regions of the world [4].

Various high-impact medical interventions effectively prevent PPH. Active management of the third stage of labor, using oxytocin as the preferred uterotonic, is prominent among them [5,6]. Administration of oxytocin, however, requires the assistance of a skilled birth attendant (SBA), and therefore is not available to women experiencing unattended home births, either by choice, lack of access to SBAs [7,8], or due to gender and wealth disparities [9-11].

Misoprostol, an oral prostaglandin E1 analogue that can be administered immediately following delivery, offers an important alternative for PPH prevention in low-resource settings and at home births, where oxytocin is not available or where its use is not feasible. Misoprostol requires no injection supplies or skilled provider for administration. Misoprostol does not need refrigeration and can therefore be stored and provided where there is no electricity. These factors enable programs for the prevention of PPH using misoprostol to potentially achieve high coverage and use, particularly by women who reside at a distance from a health facility [12-15].

Compelling evidence has emerged to demonstrate that misoprostol is both safe and effective for this indication [16-19]. This body of evidence led the World Health Organization (WHO) to amend its model list of essential medicines in March 2011 to include misoprostol for the prevention of PPH in settings “where oxytocin is not available or cannot be safely used” [20], although some have expressed concern about this decision [21].

Recently published studies have additionally concluded that the drug can be safely used at the community level through either administration by health providers [22] or distribution by community health workers (CHWs) (including traditional birth attendants [TBAs]) directly to pregnant women for self-administration at home [15,23,24]. Sutherland et al. [25] noted that this intervention is particularly cost effective. Rajbhandari et al. [23] concluded that the largest gains in protection against PPH were realized by the poor, the illiterate, and those living in remote areas.

The 2012 WHO guidelines for the prevention and management of PPH [26] have included a recommendation for the administration of misoprostol by CHWs for the prevention of PPH. The guidelines also state that, to date, there is insufficient evidence to recommend the advanced distribution of misoprostol to women for self-administration immediately after birth. A recent Cochrane review [27] noted the need for additional information concerning the feasibility of misoprostol reaching the end user (coverage), patient outcomes after use, adverse effects from misuse, and outcomes useful to policy makers, such as resource utilization. The authors of that review further urge the international community to take action to translate the research evidence about the benefits of using oral misoprostol for PPH prevention into community-based research focused on the outstanding questions about community-based distribution [28].

This integrative review of the literature was therefore undertaken to synthesize the broad array of implementation experiences and research trials (collectively called “programs”) that have used misoprostol for PPH prevention during home births. The objectives of this integrative review are 1) to describe qualitatively the program strategies for distributing and administering misoprostol for PPH prevention during home birth; and 2) where possible, quantitatively summarize the apparent success of these approaches by determining the rates of distribution, coverage (consumption by the target population), correct use, and serious adverse events associated with different distribution and administration methods. We also present additional data such as education methods and the influence that community-based distribution and use of misoprostol may have had on the trend of facility-based birth. Our selection of data is intended to emphasize those elements that we consider to be most critical to evaluating any program using misoprostol for the prevention of PPH in home births.

## Methods

### Protection of human subjects

This project was submitted to the Institutional Review Board at the Johns Hopkins Bloomberg School of Public Health, U.S.A. A notice of exempt approval was received. Project data reflected in this article were de-identified by the authors of the original reports from which information was extracted.

### Integrative review methodology

The integrative review is a comprehensive methodological approach that takes an expansive view of the type of information that can be included: it considers both qualitative or quantitative data as well as reports of both experimental and non-experimental studies [29]. The integrative review methodology widens the sampling frame beyond the limits imposed by meta-analysis (which focuses on primary studies) or systematic reviews (which focus on a single question, and place highest value on randomized clinical trials) [30]. The major limitation of integrative reviews is the potential for bias from its inclusion of non-peer-reviewed information. In addition, because integrative reviews combine information from both controlled studies and less structured data sources, fewer analytical tools are available to compare and synthesize data, leading to more qualified conclusions.

### Literature review strategy

We searched PubMed for all peer-reviewed literature published prior to December 1, 2012 using the keywords “misoprostol” and “postpartum hemorrhage” and either “home” or “community.” This information was supplemented by a web-based search of the grey literature, including non-peer-reviewed publications and project reports using the terms above. We also conducted a directed search of the websites of anticipated implementing organizations, and made inquiries among professional networks to identify unpublished information from such programs.

### Inclusion and exclusion criteria

**Figure 1:**
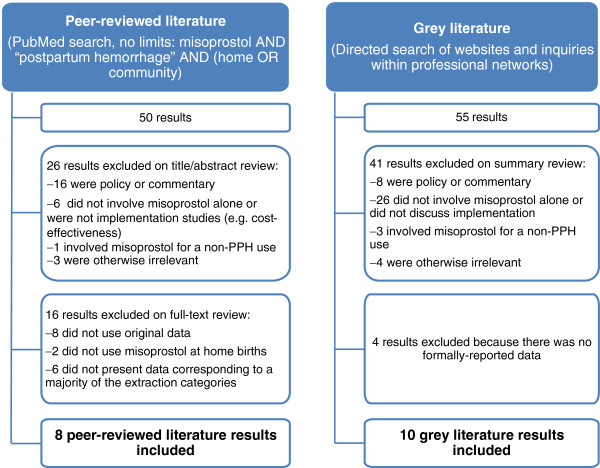
Screening and Inclusion Process.

Results from the searches and queries were first screened by a single reviewer to identify literature concerning the implementation of programs using misoprostol for the prevention of PPH. Only literature that presented final, original data regarding misoprostol use in home births and that included data that corresponded to a majority of the data elements discussed below was included for data extraction. Information that was informally shared with the study authors but that is not publicly available or available upon request to the authors in a written report was excluded. The screening and exclusion process is depicted in Figure 1.

### Data extraction

A data extraction form was developed by all authors, through an iterative process to identify all data elements that were considered most critical to the review questions. Data definitions that underpinned data extraction are presented as Table 1.

**Table 1:** Definitions

**Term**	**Definition**
Distribution Timing	The time during pregnancy when misoprostol was given to study or program participants.
Distributing Cadre	The cadre(s) of health workers responsible for giving misoprostol to women. This includes health care providers, community health workers and other community health agents, such as traditional birth attendants or community drug keepers.
Administration Method	The method by which misoprostol was administered to the women at the time of use. Typically this was administration by a health worker, administration by a community provider or self-administration by the woman or a family member.
Home Birth Rate	The national or catchment-area rate of home births as reported in the publication or written report, or the calculated proportion of home births in comparison study sites.
Administration Before Birth	Misoprostol administration while the woman is still pregnant or prior to delivery.
Adverse Maternal Outcomes	Adverse outcomes, including Maternal Death and Perceived PPH/Excessive Bleeding, that are severe and relevant to misoprostol use and that are reported as occurring in a study or program participant who delivered at home and used misoprostol.
Maternal Death	Death within 24 hours of delivery reported as occurring in a study or program participant who delivered at home and used misoprostol. Both total deaths and deaths attributed to PPH or excessive bleeding are reported.
Distribution Rate	The proportion of pregnant women in the catchment area who received misoprostol for the prevention of PPH.
Coverage Rate	The proportion of women who delivered at home in the catchment area (actual or estimated) who used misoprostol for the prevention of PPH.
Perceived PPH/Excessive Bleeding	Women’s perception of excessive postpartum bleeding or measured postpartum blood loss. A specified tool was used in some programs to measure blood loss and inform the threshold for referral.

Elements relating to program design and process included: the timing and method(s) of distribution of misoprostol; cadre(s) involved in the distribution; methods of education of distributing cadres and end-users; the person who ultimately administered the misoprostol; and methods by which the misoprostol was tracked. Elements relating to program outcomes included rates of distribution and coverage of the misoprostol, data on correct use of the drug, serious adverse events (specifically including the conduct of maternal death audits and/or verbal autopsies), and the effect on facility birth rates.

Data extraction from published studies or technical reports was conducted by two independent reviewers. The information obtained and documented from the independent data extraction processes was then compared between the two reviewers and confirmed by a third. In all cases in which there was a discrepancy of data, the issue was discussed and resolved among the authors, adhering to the wording of the original reports as closely as possible.

Information provided verbally by representatives of agencies contacted for information was cross-checked against information about the program that was available in written form. The documented information was always selected as the source of verified data. No new or secondary analysis of undocumented data was performed for this review.

Some of the information obtained concerned programs implemented by the employing agencies of this review’s authors. In these cases an independent third party reviewed all data extractions, and resolved any instances of data variance.

### Data analysis

Rates and rate ranges were computed using Microsoft Excel®. This approach was most appropriate to the nature of the data, for which traditional meta-analysis was not applicable.

Calculations of distribution (receipt) and coverage (consumption) rates required actual or estimated numbers of potential beneficiaries (for distribution, all pregnant women, and for coverage, women delivering at home) within the areas or districts forming the programs’ respective “catchment areas.” For the distribution rate, the number of pregnant women in the catchment area during the period of the intervention could be estimated by multiplying the population crude birth rate and the program’s duration.

For the coverage rate, the number of pregnant women delivering at home was estimated by multiplying the number of pregnant women in the catchment area by the program’s home birth rate. Often, the number of women taking misoprostol at a home birth was reported for only a subset of women from the study population who were followed up after delivery.

Although some programs reported several forms of incorrect use, the consumption of misoprostol prior to birth was considered most important and was reported for any program that provided this information. Analysis of adverse maternal outcomes included PPH or perceived excessive bleeding [31,32], maternal death, and other serious morbidities specifically reported by the programs. The definitions and categories used by the original authors were used wherever possible (see Table 1), so as to prevent misinterpretation or underreporting. Additional information about considerations made in selected computations is provided as footnotes to the respective tables, for the purpose of clarity and transparency.

## Results

This integrative review identified 18 programs that used misoprostol for PPH prevention among women who experienced childbirth at home (Table 2). Eight of these programs were studies with experimental or quasi-experimental designs that included comparison of misoprostol with placebo or another uterotonic. Five were operations research projects, and five were field interventions that provided misoprostol as part of a pilot or full program approach, without intention to document the clinical effect of misoprostol on PPH prevention, but, rather, to document the operational and health-related outcomes of the program’s chosen implementation methods.

**Table 2:** Characteristics of included programs

Country (* indicates peer-reviewed reference)	**Design and scope**	Home birth rate (for region or program area, where available; ^** ^**indicates national rate)**	**Number of women enrolled (for “studies,” number reflects intervention group only)**	**Number of women taking misoprostol (**^**a **^**indicates overall; **^**b **^**indicates number from postpartum subsample)**	**Administration method(s)**
Afghanistan [24]*	Study using nonrandomized experimental control design in 2 districts	80.1%	2039	1350^1^	Self
Bangladesh [33]	Operations research project in 6 districts	87%	118,594 enrolled; 77,337 delivered, of whom 53,897 received CDK^2^	46,561^a^ 1893^b^	Self and TBA
Bangladesh [14]*^3^	Study using quasi-experimental design in 2 districts	85%**	1009	884	CHW
Bangladesh [34]	Pilot project in 1 district	85%**	19,497	9228	Self
Ethiopia [13]*	Study using quasi-experimental design in 1 area	97%	500	485	TBA
Gambia [35]*	Study using randomized controlled design in 1 district	72%	630	630	TBA
Ghana [36]	Pilot project in 4 districts	37.5%	5345	1261^b^	Self
India [22]*	Study using randomized controlled design in 1 district	45.2%	812	809	SBA
Indonesia [37]	Study using nonrandomized experimental design in 2 districts	48%	1322	999	Self
Kenya [38]	Pilot project in 2 districts	38.7%	3844	1084^b^	Self and SBA
Mozambique [39]	Operations research project in 4 districts, with each of 3 sites using a different distribution strategy: 1) late ANC only, 2) TBA at birth, 3) a combination of late ANC and TBA at birth	35.3%	11,927	4781^b^	Self and/or TBA
Nepal [23]*	Operations research project in 1 district	89.1%	18,761	13,969^a^ 435^b^	Self
Nigeria [40]	Operations research project in 1 state	95%	1875	1421^b^	TBA
Pakistan [15]*	Study using randomized controlled design in 1 province	65%**	534	533	TBA
Pakistan [41]*^4^	Study using quasi-experimental design in 2 districts	61%	872	678	TBA
Tanzania [42]	Operations research project in 4 districts	30.8%	12,511	1826^b^	Self
Zambia [43]	Pilot project in 5 districts	59.9%	5574	233^b^	Self
Zambia [44]	Pilot project in 10 districts	71% (for rural areas)**	31,315	Not reported	Self and TBA

^**1**^ Administration Before Birth and Adverse Maternal Outcomes were reported for all 1421 women in the intervention group who took misoprostol, regardless of the place of delivery, but for consistency with other studies and programs (and because there was no indication to the contrary), we have assumed, particularly for the adverse outcomes reported in Table 6, that any such outcomes occurred only in those 1350 women taking misoprostol for home births.

^**2**^ Misoprostol included in CDK. The kits used by these programs included gloves, soap, a blood loss measurement mat [31,32,45] and other materials recommended for use by women who delivered at home.

^**3**^ Dose of misoprostol used was 400 μg (two tablets).

^**4**^ Misoprostol 600 μg was included in CDK.

All but one of the programs included in this review either explicitly mentioned using a dose of 600 μg misoprostol, which is commonly manufactured as three tablets of 200 μg each, or mentioned using “three tablets” and therefore presumably used a dose of 600 μg, the WHO currently recommended dosage [46]. One program used a dose of 400 μg only [14].

Thirteen of the 18 programs described their user education methods in their reports. The programs used a variety of strategies to provide information, education and communication to women and their families about the purpose and proper use of misoprostol, including individual meetings, group meetings, print media, and radio messages. Most programs emphasized the importance of delivering in a health facility as one of the key messages.

Nine programs described information on stock-outs and methods used to avoid them. All 18 specified the number of doses distributed. Accounting methods included periodic meetings among program staff (n = 8; 44.4%), stock monitoring by hand count (n = 6; 33.3%), and accounting for the voluntary return of unused drugs (n = 3; 16.7%).

Tables 3 and 4 depict the various times chosen by programs to distribute misoprostol to women, the cadres used to distribute the drug, and the individual(s) who administered the drug. Four of 18 programs (22.2%) distributed the drug earlier than 28 weeks of pregnancy. Nine programs distributed misoprostol at the time of home birth, two of which included the medication in clean delivery kits (CDKs) [45].

**Table 3:** Types of misoprostol distribution and administration

**Distribution and administration feature (multiple possible)**	**N of programs (total = 18)**	**% of programs**
**Distribution timing**		
Any ANC visit (>12 weeks)	4	22.2
Late pregnancy ANC visit (>28 weeks)	3	16.7
Late pregnancy home visit (28–32 weeks)	5	27.8
At home birth	9	50.0
**Distributing cadre**		
CHW^1^	6	33.3
TBA	7^3^	38.9
Health workers^2^/ANC providers	7	38.9
Other (family planning field worker, community drug keeper)	2	11.1
**Administration method**		
Self	11	61.1
TBA	8	44.4
CHW	1	5.6
SBA^4^	2	16.7

^1^ Includes female community health volunteers in Nepal and community-based lady health workers in Population Council’s Pakistan program.

^2^ Includes auxiliary nurse midwives in India.

^3^ One program with 99.6% CHW distribution and only 0.4% TBA distribution was considered to be CHW distribution only.

^4^ This category also includes two types of semi-skilled health workers: auxiliary nurse midwives in India and community midwives in Kenya.

**Table 4:** Distribution and coverage rates or rate ranges by distribution timing, distributing cadres and administration method (for programs for which rates were calculable)

**Distribution or administration feature (multiple possible, and for this table, the 3 Mozambique strategies are separately reported)**		**Distribution rate or rate range**	**Coverage rate or rate range**
**Distribution timing**	**Any ANC visit**	22.5–49.1%	16.8–65.9%
	**Late ANC visit**	21.0–26.7%	16.2-35.9%
	**Home visit (late pregnancy)**	54.5-96.6%	55.7-93.8%
	**At home birth**	22.5-83.6%	16.8-73.5%
**Distributing cadre**	**Community health worker**	54.5-96.6%	87.9-93.8%
	**Traditional birth attendant**	25.9-86.5%	35.9-73.5%
	**Health worker/ ANC provider**	21.0-49.1%	16.2-65.9%
	**Other**	66.5-83.6%	55.7%
**Administration method**	**Self**	21.0-96.6%	16.2-93.8%
	**Traditional birth attendant**	25.9-86.5%	35.9-73.5%
	**Community health worker**	N/A	N/A
	**Skilled birth attendant or semi-skilled health worker**	22.5%	16.8%

Health workers (including ANC providers) and TBAs were the most common distributors of the medication (7 programs each). Six programs used CHWs, and two used “other” community health personnel, such as family planning field workers or community drug keepers, in the distribution effort.

Self-administration (n = 11; 61.1%) and administration by TBAs (n = 8; 44.4%) were the two most common methods used for administration of the drug (Table 3). Additional methods included administration by CHWs and skilled or semi-skilled birth attendants.

Tables  4 and 5 illustrate the wide variation in the distribution and coverage rates achieved among the 11 programs for which sufficient information was available. Seven programs did not report sufficient information to reliably calculate either of these rates. One program in Mozambique used three different distribution strategies, resulting in similar distribution rates regardless of whether TBAs, ANC providers, or both, were the distributing cadre(s) (range of 21.0% to 26.7%); however, markedly higher coverage rates were achieved with TBAs as the distributing cadre (73.5% compared to 16.2% for ANC only). The unexpected similarity in distribution rates might be explained by the fact that only a sub-sample of women with follow-up data was included in the calculations from ANC distribution sites, while the entire sample was included in the calculations from TBA distribution sites.

**Table 5:** Misoprostol distribution and coverage rates (for programs reporting)

** Country**	**Distribution rate (%)**	**Coverage rate (%)**
Afghanistan [24]	96.6	93.8
Bangladesh [34]	66.5	55.7
Ghana [36]	49.1	65.9
Indonesia [37]	54.5	87.9
Kenya [38]	22.5	16.8
Mozambique [39]^1^		
TBA only	25.9	73.5
ANC only	21.0	16.2
TBA and ANC	26.7	35.9
Nepal [23]	72.2	Insufficient information
Nigeria [40]	83.6	Insufficient information
Pakistan [41]	86.5	Insufficient information
Tanzania [42]	26.3	29.3
Zambia [43]	40.3	Insufficient information

^1^ This program had a different distribution strategy at each of three different sites. To distinguish among approaches, results are presented for each strategy separately.

Three programs attempted to assess whether there was any change in the facility birth rate in the districts in which misoprostol was distributed for home use. In Afghanistan [24] and Zambia [43] comparison between the intervention and control areas showed an increase of 3.3% and 13.8%, respectively, in facility birth rates in the intervention areas. In Nepal [23] there was an increase of 3.9% in the facility birth rates at the end of the intervention, when compared to the beginning.

Table 6 presents the occurrence of adverse outcomes when misoprostol was used for prevention of PPH at home birth. Incorrect use of the drug (consumption before the birth) occurred in seven cases across four programs, among 12,615 users, for an overall rate of 0.06%. Many of the programs also reported instances when the drug was incorrectly administered after delivery of the placenta or if fewer than the required number of tablets had been taken.

**Table 6:** Adverse outcomes

**Outcomes**	**N of occurrences in programs reporting**^**1 **^**(total # of women taking misoprostol at home births**^**2**^**)**	**Frequency (range)**
Administration before birth	7^3^ (12,615)	0.06% (0%–0.23%)
Maternal deaths		
Total	51 (86,732)	0.06% (0%–1.72%)
Deaths due to PPH/excessive bleeding	24 (86,732)	0.03% (0.00%–0.16%)
Perceived PPH/excessive bleeding	194 (72,534)	0.3% (0%–8.9%)
Other adverse outcomes requiring hospital referral^4^	27 (86,732)	0.03% (0%–0.3%)

^1^ For Administration Before Birth and Perceived PPH/Excessive Bleeding, only those programs reporting comparable data for the specific category have been included in the calculation. For Maternal Deaths and other adverse outcomes requiring hospital referral, because of the severity of these outcomes, it has been assumed that if a study or program reported data on at least one of these outcomes and did not mention other outcomes, the other outcomes did not occur.

^2^ Some programs only collected data on these outcomes for a subsample of women taking misoprostol for home births, as noted in Table 2. The Administration Before Birth total includes subsample numbers if both overall and subsample numbers are available. The Adverse Maternal Outcomes data, however, includes overall numbers wherever available because the presence of community information sources makes it likely that such outcomes would be known and noted for the entire home-birth misoprostol population.

^3^ This includes one inferred occurrence from information that one woman in the Ghana program took misoprostol at the incorrect time and not after delivery of the placenta.

^4^ Such outcomes were enumerated in 2 programs. In one program, the outcomes were reported as including “retained placenta, postpartum eclampsia, severe lower abdominal pain, and lack of typical postpartum bleeding.” In the other program, the outcome enumerated was “severe postpartum anaemia.”

A total of 51 maternal deaths were reported among the 86,732 women taking misoprostol for home birth. A total of 24 of these deaths were attributed to perceived PPH or excessive bleeding. No deaths in the 18 programs reviewed were reported to be directly attributed to use of misoprostol.

Program reports mention three cases of suspected uterine rupture among women who took misoprostol following delivery. The diagnosis cannot be confirmed in any of these cases, given that the maternal audit methods used by these programs were not described and no autopsy was reported. The incidence of other adverse outcomes requiring hospital transfer was equal to or less than one third of 1% among 17 programs reporting on serious adverse events.

## Discussion

This integrative review shows a range of implementation approaches, data collection procedures, and documentation approaches in programs for prevention of PPH at home birth using misoprostol. We recognize the limitations in comparing programs and drawing summary conclusions from different implementation models and data reporting practices, but we believe that a sufficient number of community-level misoprostol programs have been attempted to date to render discussion and interpretation of their methods and outcomes timely and appropriate. The nature and quality of the data, a majority of which was extracted from non-peer-reviewed project reports, restricts the statistical methods that could be used in data analysis, and requires the following caveats regarding generalizability.

The information that we sought to retrieve for purposes of this integrative review was not necessarily a component of the program monitoring plans for all programs, and, even if collected, was not necessarily reported or reported in a comparable manner. As a result, there are missing or assumed data for some variables of interest. For example, a common definition of PPH as an adverse event was not present in all reports, and reports that used the term excessive bleeding were assumed to be referring to perceived PPH. Explicit mention of PPH was itself absent in one report.

Additionally, this review might be biased toward more favorable results. In addition to selective data extraction from included programs, programs that were excluded from this review because of substantial missing data might have contained unfavorable results that the implementing organizations chose not to share with the public, although this is unlikely.

It is interesting to note that a substantial number of programs did not collect or report sufficient data to estimate their distribution or coverage rates. Given that misoprostol for home birth is a strategy to achieve greater protection from PPH – regardless of location of birth – we anticipated that these data would have been more readily available.

We were particularly cautious in estimating the rates of distribution and coverage of misoprostol because we understand that most programs were not attempting to reach all pregnant women within an intervention area and did not follow up with all women who received misoprostol prior to delivery. Estimations were based on available data and assumptions regarding population or sample data. The heterogeneity of program methodologies does not allow for the formation of point estimates; therefore we present rate ranges. Footnotes in the tables present additional information about calculations. Actual distribution and coverage rates at home births could be higher than those we calculated and reported.

We present misoprostol distribution separate from its coverage because fewer women might consume the drug than those who receive it. Consumption, or coverage, presents a more accurate measure of program effectiveness than distribution because it reflects both successful distribution as well as effective counseling to the woman, her family, and any involved providers.

No particular timing was predominant among programs that distributed misoprostol prior to birth (n = 12), with programs using early, late, or unrestricted distribution timing. However, the range of distribution rates to the target population of pregnant women was lower for late ANC visit distribution compared to distribution at any ANC visit.

Programs that allowed distribution by CHWs and during home visits achieved greatest distribution and coverage, potentially more than double the coverage achieved by programs with distribution by health workers or as a part of ANC services. Distribution of the drug by other types of community-based workers also appeared to allow high distribution and coverage rates, in the very few programs for which this strategy is reported. This suggests that home-based distribution approaches, with relatively low-skilled providers, either singly or combined with facility-based approaches, can achieve high rates of distribution to the target population. This is potentially due to the pressures that health workers are under during their routine work and the difficulty that comes from adding additional tasks. CHWs, on the other hand, might be able to add this service to their work more easily, and likely have multiple opportunities to see a woman. As well, home-visit distribution by CHWs is primarily dependent on the actions of the worker, not the health-seeking behavior of the woman, whereas traditional ANC in a facility can only occur if the woman presents to the facility for care.

Eleven programs distributed misoprostol to women prior to birth. Several of these programs also allowed for administration to the woman at the time of birth at home, likely enhancing their overall distribution and coverage rates. The rates of ANC and skilled birth attendance are low in these program communities, so the programs strategically chose to provide women with protection against PPH even in situations where their births were not attended by SBAs.

Another area of great concern among maternal health advocates globally is whether a strategy of provision of misoprostol for home birth would detract from efforts at increasing facility birth rates. Only three of the 18 programs reviewed tracked this indicator. In none of those did the facility-based birth rate decline; indeed, the rate appeared to increase, although the calculation methods differ and the data do not conclusively support an attribution of changes to the programs themselves. Those three programs appeared to put a high value on education of the woman and her family regarding the importance of skilled attendance at birth, the dangers of PPH, and the use of misoprostol only for the situation where a woman is unable to achieve her plan of a facility-based birth.

The number of cases in which women took misoprostol prior to delivery is reassuringly low, as this is one of the areas of greatest concern for the international public health community. Administration before birth occurred in only seven cases out of more than 12,000 women who were followed up (0.06%). One case was due to a woman taking the dose before delivery of a second twin. The second twin delivered normally without complication. Another case was a woman responding to a domestic dispute with intention of self-harm. She was immediately identified and referred to a nearby facility where she delivered normally within 12 hours. Authors reporting on the Ghana program stated that there were four women who took the drug at the wrong time, three of whom took the drug after delivery of the placenta. We therefore assume that the fourth case was that of a woman who took the drug prior to birth, but no further information is available from the program description. Four cases occurred in one large program in Bangladesh for which there was no specific information about circumstances or outcomes. It is possible that there might be additional cases of administration prior to the birth that were unreported, although the likelihood of this is low, given the high profile of most of these programs.

With such a low occurrence of premature administration, it is difficult to draw any meaningful distinctions among the programs, each of which had various and unique features in design. More of the cases of premature administration occurred when the drug was distributed at any ANC visit compared to ANC or home distribution closer to the time of birth, and when distribution was by a health worker or ANC provider compared to distribution by a lay health worker.

All but one program made an attempt to identify and record the number of maternal deaths in the program’s target area, and specifically, the number of maternal deaths that occurred among women who took misoprostol. Virtually every program that recorded the number of maternal deaths also noted the method(s) by which the deaths were investigated. Investigations were also commonly undertaken to verify accounts of reports of excessive postpartum bleeding reported by women, their family, or their birth attendants. Such rigorous methods help ensure that such deaths can be more independently reviewed and evaluated for any relationship to either the drug or its method of distribution or administration. It is reassuring that there were no cases of maternal death that were attributed to misoprostol across the almost 87,000 women who took the drug as part of these programs.

## Conclusion

This integrative review has synthesized the available body of information about completed programs using misoprostol for prevention of PPH at home birth. The quantity and comparable quality of available data are limited, and the non-peer-reviewed sources of the majority of these data restrict the rigor of the statistical approaches used for data analysis. However, even given these limitations, findings from this review should promote understanding about the outcomes of various misoprostol program approaches and begin to address outstanding concerns by describing the outcomes of program outreach.

Findings from this review of 18 independent programs conducted in 14 low-resource countries qualitatively demonstrate that it is possible to achieve high distribution and coverage of misoprostol especially when community health systems are engaged in the distribution effort. Programs that distributed misoprostol at home visits late in pregnancy or at the time of birth, as well as those that used community-based personnel, appear to achieve higher coverage than those that used formal health workers and ANC distribution, either alone or in combination with home distribution.

Self-administration by the woman and administration by the TBA have been the most common methods of administration of the medication, and programs that used these administration methods achieved higher coverage rates than those that required skilled or semi-skilled birth attendants for administration. Programs that educate women and families for self-administration of misoprostol appear to be safe, with an extremely low rate of erroneous early administration.

While few programs provided data on changes in facility birth rates, and none permit attribution of those changes directly to the misoprostol distribution efforts, community-based programs using misoprostol at home births do not appear to work against national efforts to increase facility birth rates. Future misoprostol programs should be designed in a manner that ensures adequate and comparable data collection regarding the key features and outcomes discussed in this review, namely, distribution, coverage, correct use, education, and effect on facility birth rates.

## Abbreviations

ANC: Antenatal care;CDK: Clean delivery kit;CHW: Community health worker;PPH: Postpartum hemorrhage;SBA: Skilled birth attendant;TBA: Traditional birth attendant;WHO: World Health Organization

## Competing interests

JMS, RG, NP and MMH, are current or former employees or consultants of Jhpiego or Venture Strategies Innovations. These organizations have been involved for many years in implementation of programs to reduce PPH at home birth using misoprostol throughout Africa and Asia.

## Authors’ contributions

JMS and NP conceived of the study and participated in its design and coordination. RG and MMH conducted the literature search and data extraction. All authors conducted analysis and developed the findings. JF contributed to the writing of the manuscript. All authors read and approved the final version of the manuscript.

## Pre-publication history

The pre-publication history for this paper can be accessed here:

http://www.biomedcentral.com/1471-2393/13/44/prepub
